# Design, Synthesis and Characterization of Vitrimers with Low Topology Freezing Transition Temperature

**DOI:** 10.3390/polym14122456

**Published:** 2022-06-16

**Authors:** Baiju P. Krishnan, Kay Saalwaechter, Vico K. B. Adjedje, Wolfgang H. Binder

**Affiliations:** 1 Macromolecular Chemistry, Division of Technical and Macromolecular Chemistry, Institute of Chemistry, Faculty of Natural Sciences II (Chemistry, Physics and Mathematics), Martin Luther University Halle-Wittenberg, von-Danckelmann-Platz 4, D-06120 Halle, Germany; baijupkrish@gmail.com (B.P.K.); vico.adjedje@chemie.uni-halle.de (V.K.B.A.); 2 Institut für Physik—NMR, Martin-Luther Universität Halle-Wittenberg, Betty-Heimann-Str. 7, 06120 Halle (Saale), Germany; kay.saalwaechter@physik.uni-halle.de

**Keywords:** vitrimers, recyclable, self-heal, polymers

## Abstract

Vitrimers are crosslinked polymeric materials that behave like fluids when heated, regulated by the kinetics of internal covalent bond-exchange that occurs rapidly at or above the topology freezing transition temperature (*T_v_*) of the vitrimer, making these materials readily reprocessable and recyclable. We report two novel multiphase vitrimeric materials prepared by the cross-linking of two polymers, namely poly(triethylene glycol sebacate) and poly(2-hydroxyethyl acrylate), using zinc acetate or tin(II) 2-ethylhexanoate as catalysts, which exhibit significantly low *T_v_* temperatures of 39 °C and 29 °C, respectively. The transesterification reactions allow rapid and pronounced stress relaxation at high temperatures, following the Arrhenius law. The lower *T_v_* of these vitrimers could be attributable to the flexible long chains of these polymers and the significant excess of OH moieties present along the main chain of the polymer. The design of such multiphase vitrimers is not only useful for the practical application of vitrimers to reduce plastic waste but could also facilitate further development of functional polymer materials that can be reprocessed at low temperatures.

## 1. Introduction

Covalent adaptable networks (CAN) that contain exchangeable reactions are not only useful to induce deformability (reprocessibility), but they also feature unique self-healing functions [1,2,3,4,5]. So-called dissociative CANs, based on the dissociation and reforming of a network via stimuli-activated reversible reactions, such as the Diels–Alder reaction, result in depolymerization to oligomers or monomers and, thus, finally in the loss of their network integrity and increasing solubility in processing solvents [6,7]. In contrast, approaches based on associative pathways, in which both occur simultaneously (bond formation and bond breaking), result in stimuli-induced deformation of the network without the loss of material properties, which represents an equally attractive approach. It is well known that crosslinked polymers, exhibiting stress relaxation without a concomitant change in material properties, can be prepared via associative CAN by photoaddition fragmentation chain transfer reactions of allyl sulphides [8,9] or trithiocarbonates [10,11,12]. Although these CANs were very exciting, applications were limited due to the lack of stability of the radicals involved during their topology rearrangements.

Leibler and co-workers developed associative CAN networks composed of the diglycidyl ether of bisphenol A and a mixture of tri- and dicarboxylic acid compounds that behave like a thermoset polymer at room temperature and are deformable at higher temperatures [13]. The rapid topology reorganization, such as with epoxy/acid or epoxy/anhydride networks, is due to the thermally activated intermolecular transesterification between ester and alcohol groups in the presence of a transesterification catalyst. The viscosity of these polymeric materials gradually decreases with temperature as the rate of intermolecular transesterification increases following the Arrhenius law, which is a typical property of vitreous silica; hence, these polymers are called vitrimers [14]. This pioneering work on vitrimers and the subsequent reports have motivated many other research groups to design and synthesise various vitrimers using not only transesterification reactions [15,16,17,18] but also other dynamic covalent chemical processes, such as transaminations [19,20,21], transalkylations [22,23,24,25,26], siloxane-silanol exchange [27,28], olefin-metathesis exchange [1,29], disulfide exchange [30,31,32], and conjugate addition-elimination reactions [33]. The studies of such bond reorganization allows us to gain mechanistic insights [1] and specific applications such as shape memory behaviour [27].

Vitrimer reprocessability relies on rapid topology reorganization due to exchange reactions triggered by thermal stimuli. Besides the glass transition temperature (*T_g_*) and the melting point (*T_m_*), which are the most important temperatures that determine the service temperature of common polymers, there is another important temperature for vitrimers—the topology freezing transition temperature (*T_v_*), which indicates the upper service temperature and lower reprocessing temperature of a vitrimer [13]. Above *T_v_*, a more rapid exchange reaction and topology facilitates recycling, whereas below *T_v_* it resembles a conventional thermoset because the exchange reaction is slow. Since *T_v_* has a direct impact on the performance and recycling of vitrimers, it is necessary to understand the factors that affect *T_v_* of a vitrimer [18,34]. The *T_v_* of vitrimers can be most directly controlled by changing the content or type of catalysts [17,35]. With a higher catalyst content, a lower *T_v_* of a vitrimer can usually be obtained. Since different catalysts generate different activation energies, networks prepared with different catalysts will display different *T_v_* values [35]. Another way to control *T_v_* is to introduce flexible linkers into the network. Helms and co-workers reported that the *T_v_* decreases with increasing molecular weight of the flexible linear segments, but increases with increasing molecular weight of the rigid segments, because the reconfigurability of vitrimers is affected not only by the thermodynamics/kinetics of bond exchange at a given network density, but also by the mobility of the polymer chains within the network [36]. We anticipate that it would be possible to design low-*T_v_* vitrimers if two flexible polymers chains are introduced as components of a polymer network. We herein report low-*T_v_* copolymer networks of flexible poly(trethylene glycol sebacate) (**1**) and flexible poly(hydroxyethyl acrylate) (**2**) that exhibit vitrimeric behaviour due to a transesterification reaction between the ester groups of Polymer **1** and the excess hydroxyl groups of Polymer **2** in the presence of transesterification catalysts such as zinc acetate (Zn(OAc)_2_) and tin(II) 2-ethylhexanoate (Sn(Oct)_2_) [13].

**Figure 1:**
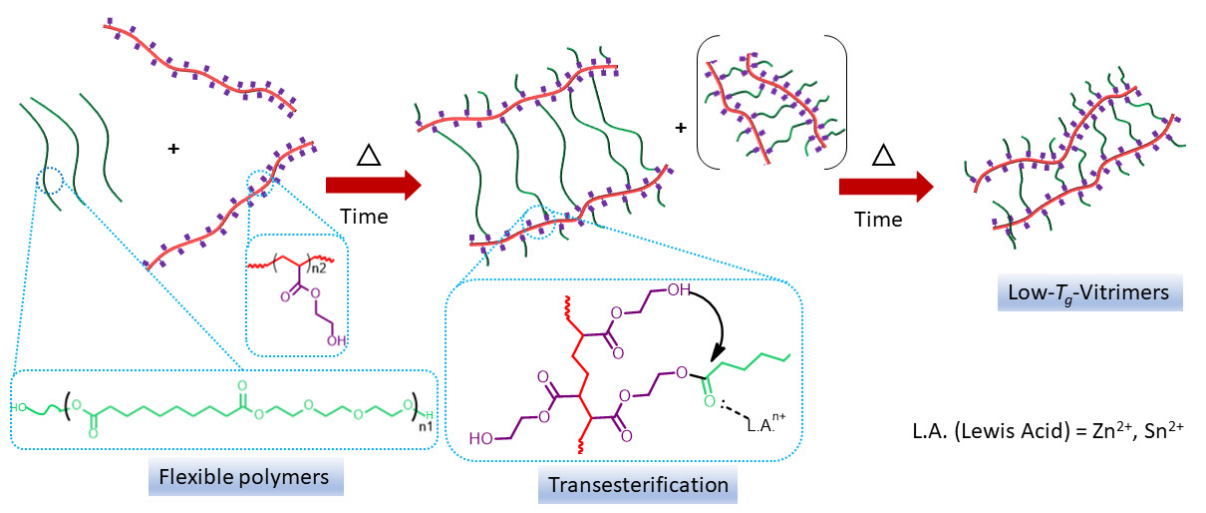
The proposed molecular structure and possible transesterification reactions of the investigated vitrimers. The triangle symbol (Δ) represents heating.

The involved chemical structures and the concept of our new material are illustrated in Figure 1. The main idea is that we expect that the high abundance of dangling OH groups of the PHEA (MW of about 9 kDa) will lead to a particularly fast transesterification and, thus, to a low *T_v_*. Another aspect to consider is that the ester linkages in the polyester (**1**) (PTGS) with a MW of around 3 kDa—thus displaying about 9 repeat units—must be expected to participate in the transesterification reactions. Therefore, at long times as shown on the right, we expect that an equilibrium structure is formed that will consist of rather short triethylene glycol- sebacic acid (TGS) links between the PHEA chains (possibly just one sebacic acid and two triethylene glycol units), and that the final structure has many dangling oligomeric PTGS chains. These chemical structures involved in the designed vitrimers could help to transform a loosely interconnected network into a more interconnected compact network as transesterification proceeds, equipping the vitrimer with a more rigid and better-defined shape. The data presented in this paper are consistent with this expectation.

## 2. Materials and Methods

### 2.1. Materials

Chemicals and solvents were purchased from Sigma–Aldrich, TCI Chemicals, and Alfa Aesar and used without further purification. Prior to polymerization, azobisisobutyronitrile (AIBN) was purified by recrystallization in methanol. Approximately 2 g of AIBN were weighed and transferred to a capped Erlenmeyer flask, along with approximately 50 mL of methanol. The mixture was stirred at room temperature for 1 h and then the solution was filtered, after which the undissolved portion was discarded while the filtrate was placed in a refrigerator for 24 h (5 °C). The crystals formed were filtered, dried and stored in a glass bottle. 2-Hydroxyethyl acrylate (HEA) was passed through a basic alumina column prior to polymerization to remove the radical inhibitor.

### 2.2. Characterization

**Differential Scanning Calorimetry** (DSC) measurements of vitrimers were performed using a Netzsch DSC 204 F1. Polymer samples with a mass of 2–5 mg were placed in aluminium crucibles and heated under a nitrogen atmosphere at a heating rate of 10 K/min. The temperature range of experiments was −70 °C to 150 °C. Data analysis was performed using Netzsch Proteus Analytic software.

**High-resolution nuclear magnetic resonance** (NMR) spectra were recorded using a Varian Gemini 400 spectrometer (Agilent Technologies Co., Santa Clara, CA, USA) with deuterated chloroform (CDCl_3_) or DMSO-d_6_ as solvent. MestReNova software (version 9.0.1-13254) was used to analyse the NMR data.

**Fourier transform infrared spectroscopy** (FTIR) of the polymer samples was performed using a Bruker Tensor VERTEX 70 spectrometer equipped with a Golden Gate Diamond ATR top plate in an attenuated total reflection (ATR) mode. FTIR spectra were recorded between 4000 and 400 cm^−1^. Opus 6.5 was used to analyse the data.

**Gel permeation chromatography** (GPC) studies were performed using a Viscotek GPCmax VE 2001 (ViscotekTM) with HHR-H-Guard-17369 and GMHHR-N18055 columns and in DMF solution containing 10 mM LiNTf_2_ at a flow rate of 1.0 mL min^−1^. External calibration was performed using polystyrene standards, and data were analysed using OmniSEC software (v 4.5.6).

**Rheology** experiments were performed on an Anton Paar MCR 101 with a parallel plate geometry using 8 mm sample disks (thickness 200 µm–400 µm). Unless otherwise specified, experiments were performed with a normal force of 10 N, an oscillation frequency of 6.28 rad/s and a strain of 2%. For all rheological experiments, the applied stress was in the linear viscoelastic range at the measured temperatures. For amplitude sweep experiments, the strain was varied between 0.01% and 100%. In frequency sweep experiments, the angular frequency varied from 1 to 100 rad/s, and the storage modulus (*G′*) was followed over time at a constant temperature. In stress relaxation experiments, a strain of 2% was applied to the material, and the relaxation modulus (*G*(t)) was tracked over time at constant temperature. *T_v_* of the vitrimers was calculated according to the literature [17,35,37]. In all brevity, *T_v_* is the temperature at which the viscosity reaches 10^12^ Pa•s. *η*(*T*) is obtained from the measured data via the Maxwell relation, *η*(*T*) = *G τ* ^∗^(*T*), where *τ* ^∗^(*T*) is obtained from stress-relaxation tests.

**Swelling tests** were performed by immersing 20 mg of the vitrimer in 2 mL of THF for 48 h at 25 °C. The solvent was then removed and the sample was dried under reduced pressure overnight at 100 °C. The swelling ratio was calculated from the initial mass and the swollen mass of the network, and the soluble fraction was measured from the initial mass and the dried mass of the network according to the data in the literature [38].

**Electrospray Ionization Time-of-Flight mass spectrometry** (ESI-TOF-MS) measurements were performed on a Bruker Daltonics microTOF via direct injection at a flow rate of 180 μL h^−1^ in the negative mode with an acceleration voltage of 4.5 kV. Samples were prepared by dissolving in LC-MS grade methanol. The instrument was calibrated using the ESI-L low concentration tuning mix from Agilent Technologies (product no. G1969-85000). The software Data Analysis (version 4.0) was used for data evaluation.

**Proton low resolution NMR experiments** were performed on a Bruker minispec mq20 low-field NMR spectrometers according to the previous published procedures [39,40,41,42]. Phase distinction in static proton NMR is based on the effect of molecular motion (mostly segmental rotations) on the spectral linewidth, or, as done here, without Fourier transform in the time domain on the directly detected free-induction decay (FID). We further used a suitable refocusing pulse sequence, termed magic-sandwich echo (MSE), in order to overcome the receiver dead-time problem [43]. Secondly, we applied multiple-quantum (MQ) NMR for characterization of the network structure [42,44,45].

### 2.3. Synthesis of Poly(Trithylene Glycol Sebacate) (***1***)

Sebacoyl chloride (4.46 mL, 20.9 mmol) was added dropwise to the triethylene glycol monomer (3.57 mL, 26.1 mmol) under a nitrogen atmosphere for 30 min at 80 °C, and then the mixture was stirred magnetically overnight. The obtained gummy product was washed three times with dry diethyl ether to remove unreacted monomers and dried under reduced pressure, yielding 5.01 g of **1** (76%) [46]. ^1^H NMR (CDCl_3_, 400 MHz) δ: 4.24–4.20 (m, 4H), 3.74–3.71 (m, 1H), 3.70–3.67 (m, 4H), 3.66–3.65 (m, 1H), 3.64 (s, 4H), 3.60–3.59 (m, 1H), 2.33–2.29 (m, 4H), 1.63–1.57 (m, 4H), 1.29 (s, 8H). ^13^C NMR (CDCl_3_, 100 MHz) δ: 173.72, 72.45, 70.56, 70.53, 70.36, 69.24, 69.20, 63.29, 63.20, 61.18, 34.14, 34.12, 29.07, 29.05, 29.03, 24.83, 24.82.

### 2.4. Synthesis of Poly(Hydroxyethyl Acrylate) (***2***)

Free-radical polymerization of 2-hydroxyethyl acrylate (HEA) was used for the synthesis of polymer **2** [47]. HEA (6 g, 51.7 mmol), DMF (18 mL) and AIBN (335 mg, 2.0 mmol) were added to a Schlenk flask. After three freeze-thaw pump cycles, the flask was heated to 70 °C in an oil bath. Polymerization was carried out under an atmosphere of argon and via magnetic stirring for 3 h. After 3 h, an excess amount of acetone was added and a colourless sticky solid was obtained, which was then washed again several times with acetone and dried under reduced pressure to obtain 4.91 g of colourless sticky solid (82%). ^1^H NMR (DMSO-d6, 400 MHz) δ: 4.75–4.69 (m, 1H), 3.99 (broad, 2H), 3.55–3.53 (m, 2H), 2.48 (broad, 1H), 1.80–1.58 (m, 2H). ^13^C NMR (DMSO-d6, 100 MHz) δ: 174.63, 66.11, 59.29, 31.21, 22. 49.

### 2.5. Synthesis of the Vitrimers ***3*** and ***4***

Polymers **1** (1.0 g, 0.35 mmol) and Polymer **2** (1.0 g, 0.11 mmol) were placed in a small Petri dish and mixed thoroughly at 50 °C. To this mixture was added, with stirring, a methanolic solution of Zn(OAc)_2_ (6 mL of 71.76 millimolar) (0.43 mmol). The amount of catalyst was calculated to be 5 mol%, based on the repeating unit of Polymer **2**. The molar ratio of [OH]/[COOR] was equal to 1/1.53. After the mixture was well mixed, the Petri dish was heated at 80 °C for 3 h to remove the methanol and then kept in an oven at 170 °C for 6 h to obtain Vitrimer **3**. In the case of Vitrimer **4**, Sn(Oct)_2_ was used as a catalyst (139 µL, 0.43 mmol), which was added to the mixture of **1** and **2** without solvent. The Petri dish was then kept in an oven at 120 °C for 6 h to obtain Vitrimer **4**.

## 3. Results and Discussions

Low-*T_g_* polymers are an important prerequisite for the development of low-*T_v_* vitrimers because they increase the degree of freedom of the conformation of the flexible chains in the vitrimer network [36]. Poly(hydroxyethyl acrylate) is a low-*T_g_* polymer and contains abundant nucleophilic hydroxyl groups that potentially can trigger transesterification [48]. The long-chain polyesters not only display a low-*T_g_*, but can also act as multiple nucleophilic centres of transesterification, which increases the probability of exchange reactions in the vitrimeric network.

**Figure 2:**
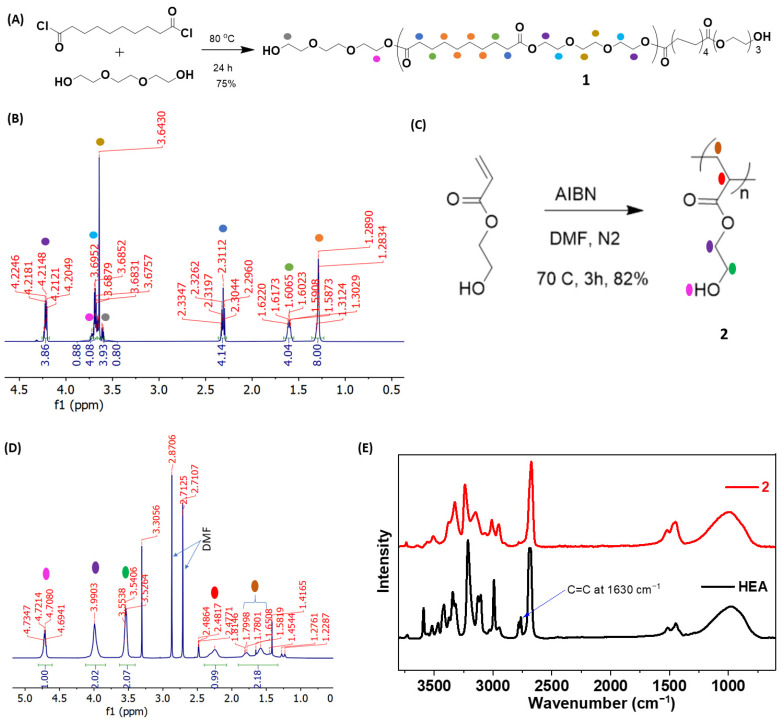
(**A**) Bulk polymerization of sebacoyl chloride and triethylene glycol at 80 °C. (**B**) ^1^H NMR spectrum of Polymer **1**. (**C**) Free radical polymerization of HEA monomer. (**D**) ^1^H NMR spectrum of Polymer **2.** (**E**) Comparison of FTIR spectra of HEA monomer and Polymer **2**.

Polymer **1** was synthesized via bulk polymerization by slowly adding sebacoyl chloride to a slight excess in molar ratio of triethylene glycol at 80 °C to synthesize polyesters with triethylene units (Figure 2A). GPC analysis showed a polydispersity of 1.39 and a molecular weight of 2.85 kDa (Figure S1A, Supporting Information). ^1^H NMR clearly indicaed the presence of terminal CH_2_ protons attached to hydroxyl groups at *δ* 3.60 ppm (Figure 2B and Figure S2A, Supporting Information). In addition in ^13^C NMR only one kind of C=O signal was detected, belonging to the ester carbonyl group in Polymer **1**, proving the absence of terminal carboxylic acid moieties in Polymer **1** in amounts of <10 mol% (Figure S2B, Supporting Information). The amount of hydroxyl groups was measured to be 3.31 × 10^−4^ mol/g, which was determined from ^1^H NMR by comparing the relative intensities of the backbone signal at *δ* 4.22 ppm and the end groups at *δ* 3.60 ppm [49]. These additional hydroxyl groups help in the crosslinking process with polymer **2**. Polymer **2** was synthesized by free-radical polymerization using AIBN as the radical initiator (Figure 2C). The polymerization was confirmed by NMR and FTIR experiments. ^1^H NMR clearly showed the disappearance of the signal between 5.5–6.5 ppm, indicating the consumption of alkene protons bonds by the polymerizations (Figure 2D and Figure S3, Supporting Information). FTIR analysis also confirmed that the C=C bond stretching band of HEA monomer disappeared in Polymer **2** (Figure 2E). In GPC analysis the polymers had a broad distribution with an average Mn of 9.45 kDa (Figure S1B, Supporting Information), as usually observed in the free radical polymerization of HEA monomers [50].

**Scheme 1:**
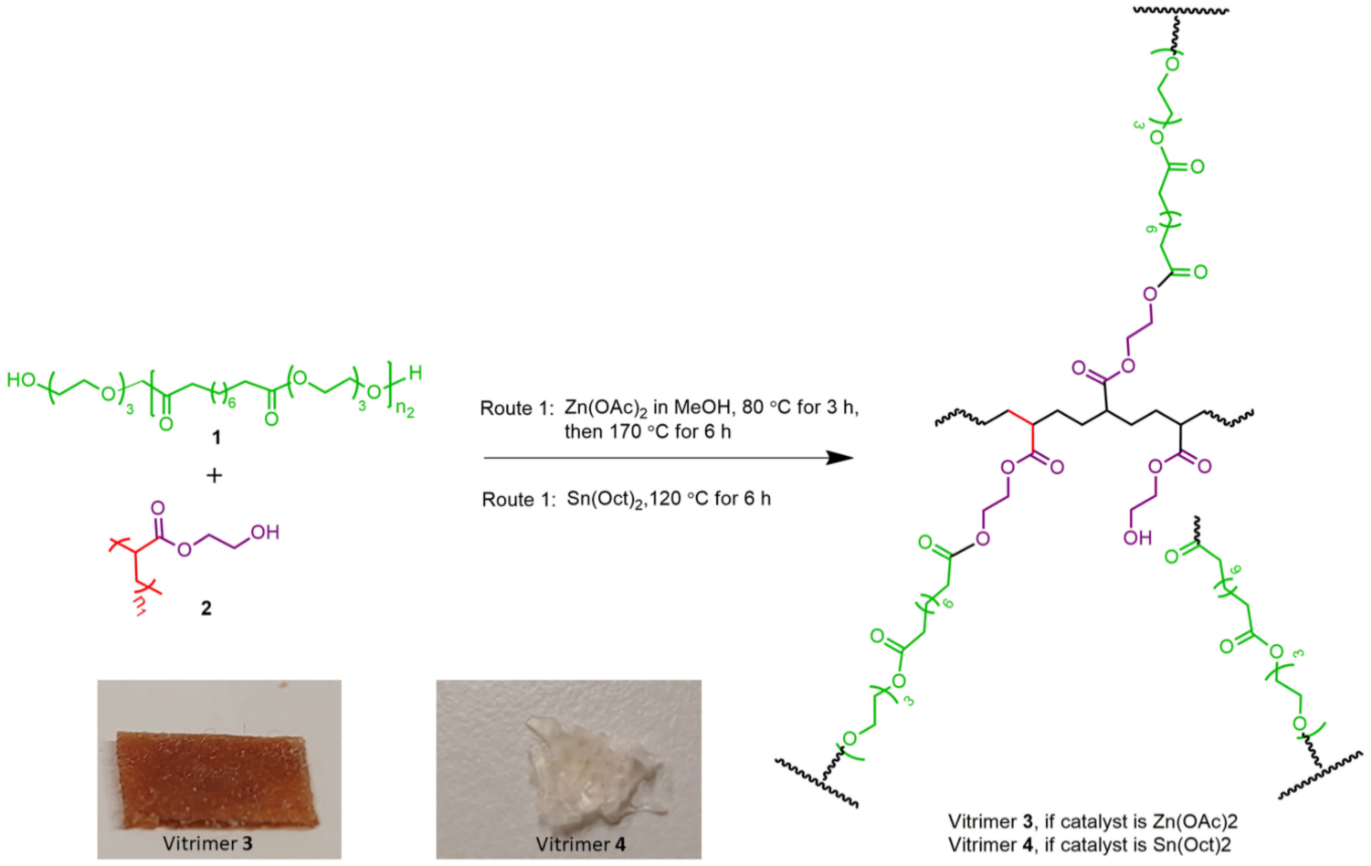
Synthesis of the Vitrimers **3** and **4** and their physical appearance (see photos).

Two sets of copolymer networks were synthesised using Polymer **2** (9.45 kDa) as a hydroxyl precursor and Polymer **1** (2.85 kDa) as a crosslinking agent in the presence of a catalyst with a concentration of 5 mol%, based on the monomer units of polymer **2** (Scheme 1). The resulting networks (20 mg) were immersed in THF (2 mL) for 48 h at room temperature to measure the swelling ratio and the soluble fraction. Vitrimer networks **3** and **4** showed identical swelling behaviour in THF (160–170%) and the mass loss of both vitrimers was limited to only ≤10 wt%, indicating that crosslinking is efficient in these materials. The networks were dried under reduced pressure and subsequently their mechanical and vitrimeric properties were investigated on thin films of these networks. From ^1^H NMR and ESI-TOF-MS of the soluble fraction of Vitrimer **3**, it is also evident that ethylene glycol units cleaved from Polymer **2** reacted with Polymer **1**, indicating the possibility of transesterification reactions between Polymers **1** and **2** (Figures S4 and S5, Supporting Information).

**Figure 3:**
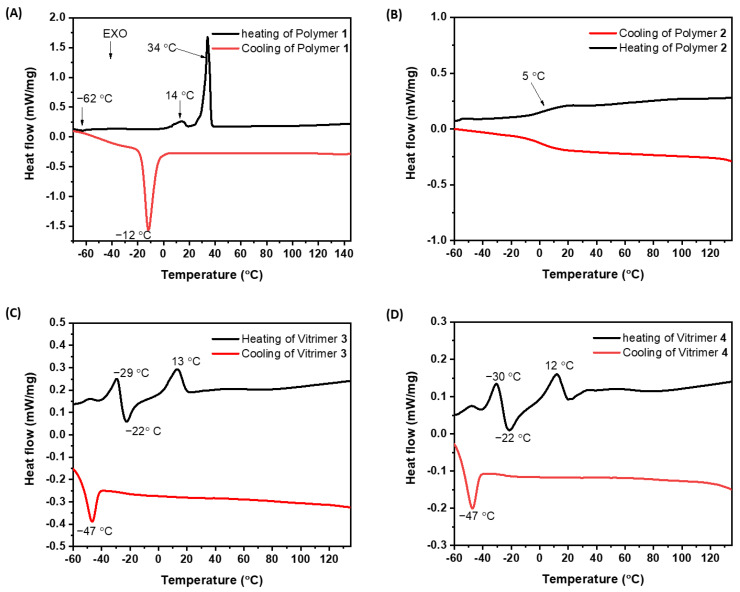
DSC profiles of the polymers **1** (**A**) and **2** (**B**) and the vitrimers **3** (**C**) and **4** (**D**) (temperature scan at a rate of 10 Kmin^−1^).

The thermal behaviour of the vitrimers **3** and **4** was almost identical, although the properties of the starting polymers are quite different (Figure 3). Polymer **1** displays a low *T_g_* at −65 °C and showed two transitions at 14 °C and 34 °C, the first due to an irreversible polymorphic transition and the second due to melting of the polymer, indicating its semi-crystalline nature (Figure 3A). In contrast, Polymer **2** is mainly amorphous and has a *T_g_* of 5 °C (Figure 3B). After crosslinking of the polymers **1** and **2**, the thermal properties of the vitrimers as determined by DSC became more complex and contained multiple melting peaks at −29 °C, 13 °C and crystallization peaks (−22 °C in heating and −47 °C by cooling) in the network (Figure 3C,D). This could be due to the fact that in crystalline-amorphous block copolymers, a higher order structure can be formed by the combination of crystallization and microphase separation (MS) when the block copolymer is crystallized from microphase-separated melts [51]. We hypothesize that these multiple melting and crystallization peaks in the DSC profiles may be due to the presence of two types of higher-order structures in our vitrimeric systems.

**Figure 4:**
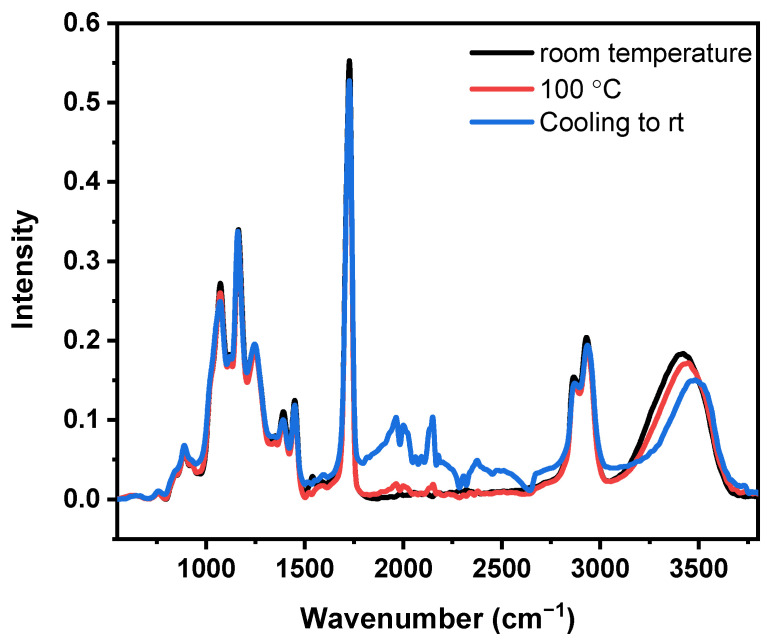
A comparison of the FTIR spectra of Vitrimer **4** at room temperature, 100 °C and cooling to room temperature after 100 °C.

The low-temperature component melts at −29 °C and recrystallizes to an intermediate structure, which subsequently melts at 13 °C. It was found that the intermediate structure is not observed upon cooling, indicating that it can only be obtained through the melting of the low-temperature structure (Figure 3C,D). In addition, we observed a broad endothermic peak between 30–80 °C, which could be due to a change in the hydrogen bonding pattern of poly(2-hydroxyethyl acrylate) upon heating, which is already known for the homopolymers of poly(2-hydroxyethyl methylacrylate) [52]. The temperature-dependent FTIR analysis of Vitrimer **2** clearly shows that the signal from OH was blue-shifted from 3059–3660 cm^−1^ with a peak value of 3413 cm^−1^ to 3224–3693 cm^−1^ with a peak value of 3478 cm^−1^ at 100 °C (Figure 4). When the heated sample was cooled to room temperature, the original hydrogen pattern almost reversed. However, the slight change after cooling to room temperature could be due to the microphase separation in our vitrimers at room temperature, which causes a longer relaxation to reach its original hydrogen-bonded structure.

**Figure 5:**
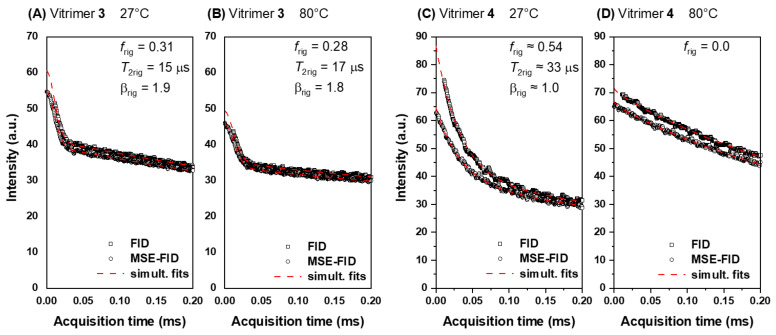
The free-induction decay (FID) and MSE-refocused FID analyses of Vitrimer **3** (**A**,**B**) and Vitrimer **4** (**C**,**D**) at different temperatures.

Microphase separation in our vitrimeric systems is further confirmed by the joint analysis of free-induction decay (FID) and MSE-refocused FIDs at different temperatures (Figure 5). We detect and thus “count” protons in the sample, which allows for an objective quantification of phases with different mobility [41,42]. Note that these experiments were done at room temperature and above; thus, we focus on the range above the different melting transitions seen in DSC. The data shown in Figure 5 could be fitted with a superposition of only two fractions modeled with stretched/compressed exponential functions, $FID_{i}=A*f_{i}*\exp\left[-{\left(\frac{t}{T_{2i}}\right)}^{\beta_{i}}\right]$, where A is an amplitude factor, *T*_2*i*_ the relaxation time and *β*_i_ the stretching exponent ranging from below 1 (distributed exponential relaxation) to 2 (Gaussian decay). We have simultaneously fitted the FID and the MSE-refocused FID signal to accurately determine the transverse relaxation time (*T_2_*) and β parameters for the most rigid component subject to the dead time problem. As one can appreciate, some of the signal is lost during the MSE, and this loss is a measure of the timescale of molecular motion within that phase [41]. It is maximal when the correlation time is of the order 10 µs, which is usually the case at around 40 K above *T_g_*.

Between room temperature and 80 °C, Vitrimer **3** has a 30% rigid component with a *T_2_* decay of about 17 us and an exponent *β* of around 2, corresponding to a Gaussian signal typical for rigid solids. It coexists with a liquid/mobile component with a *T_2_* being one to two orders of magnitude longer. While semi-crystalline polymers often display a similar signature, we noted that the component gradually softens at around 180 °C (Figure S6, Supporting Information), where the signature is similar to Vitrimer **4** at a much lower temperature (27 °C). Thus, this rigid component has a *T_g_* of the order of 140 K. We suspect that it corresponds to rather tightly (over)cross-linked regions, e.g., diols directly connecting PHEA chains. This suggests that Vitrimer **3** is rather inhomogeneous. In contrast, Vitrimer **4** has about 50% (subject to a larger fitting uncertainty) of a component with a *T*_2_ of about 33 us and a near-exponential decay (β around 1). Thus, it is not completely rigid but just within the softening region (about 40K above its *T_g_*) at 27 °C, i.e., similar to Vitrimer **3** at 180 °C. At 80 °C, this rigid component is already no longer visible, i.e., it is mobilized and thus elastomeric. This sample is thus more homogeneous and closer to the “equilibrated vitrimer” shown in Figure 1 on the right. Overall, these decay data clearly show that our vitrimers exhibit microphase separation at room temperature and that Vitrimer **4** becomes dynamic and more homogeneous upon heating compared to Vitrimer **3**.

**Figure 6:**
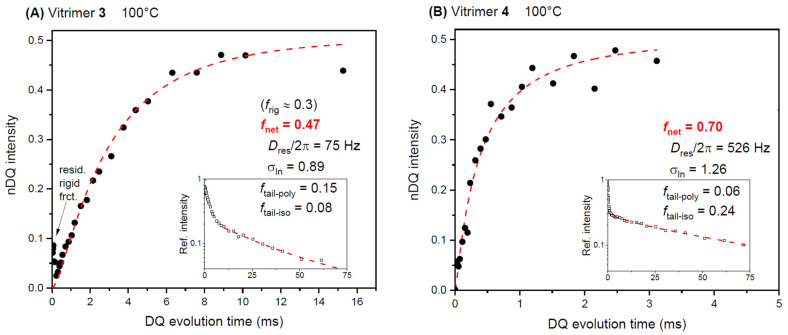
MQ NMR for characterization of the network structure of Vitrimers **3** (**A**) and **4** (**B**). The symbols are data and red dashed lines are the tits.

To shed more light into the cross-linked network structure, we have applied MQ NMR. The experiment provides two different data sets, the reference and the double-quantum (DQ) intensity measured as a function of pulse sequence duration (DQ evolution time), subject to further processing [42,44,45] (Figure 6). In the first step, shown in the insets, a “tail” with long *T_2_* relaxation time is identified. It quantifies a fraction of chains that are more isotropically mobile and thus do not carry a load in the network, i.e., defects. We distinguish more constrained (long-chain polymeric) defects and fast-moving fully isotropic components (short dangling ends and sol) that have a *T_2_* differing by a factor of 5–10. In both cases, these two fractions comprise 25–30% of the samples, where Vitrimer **4** has more highly mobile (possibly dangling) defects, in agreement with the structure shown in Figure 1 on the right. Vitrimer **3** features more of the less mobile long-chain defects, indicative of a structure where longer PTGS chains have survived (Figure 1, middle).

In the second step, the tail-corrected reference intensity is used to remove relaxation effects from the DQ intensity, resulting in normalized DQ (nDQ) build-up curves. These curves reflect the cross-linking density, given in an “NMR unit” termed residual dipolar coupling (*D*_res_) measured in Hz. It characterizes the anisotropy of segmental motions induced by the fixed chain ends, which increases when network chains are shorter. The spatial homogeneity of the network is also encoded in the shape of the curve [45]. The standard deviations of assumed lognormal distributions are of an order of unity, which means that both networks are topologically quite inhomogeneous. The most notable difference is the large contrast between *D*_res_ of the vitrimers, where Vitrimer **4** has an approximately 7 times higher cross-link density (7 times shorter network chains).

This last result, and in fact all NMR results, are again in good agreement with the sketch in Figure 1, based on the assumption that Vitrimer **3** is dominated by longer PTGS chains linking the PHEA, while in Vitrimer **4** the now different catalyst has aided in also attacking the ester linkages in the PTGS, thus obtaining a more equilibrated structure with shorter oligomeric PTGS linkages between the PHEA chains. Obviously, transesterification of the different ester groups in the structure occurs on different timescales or with different efficiency depending on the chemical surroundings. Finally, we summarize that Vitrimer **3** has about 30% of quite rigid over-crosslinked regions embedded in a weakly cross-linked matrix comprising ca. 50% of the sample (where the former possibly act as reinforcing filler), while Vitrimer **4** is more homogeneous, in the sense that it has a (in itself still topologically heterogeneous) more highly cross-linked network structure and about 30% of low-molecular dangling structures. As suggested by the DSC results, phase separation and partial crystallization of the PTGS units is an issue in both samples at temperatures below 20 °C.

**Figure 7:**
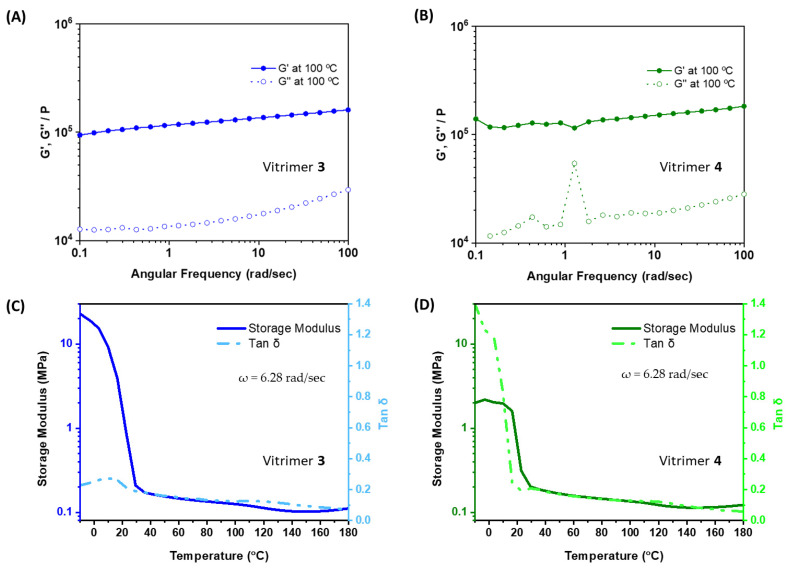
An investigation of storage and loss moduli upon frequency sweep on vitrimers **3** (**A**) and **4** (**B**), as well as G′ and Tan δ vs. temperature of vitrimers **3** (**C**) and **4** (**D**).

Although our vitrimer networks **3** and **4** exhibit microphase separation at room temperature and become more homogeneous at higher temperatures (above 150 °C), we expect vitrimeric behaviour at these temperatures. Frequency-sweep measurements of vitrimers **3** and **4** in the region of the wide rubbery plateau show that the storage moduli (G′) are significantly higher than the loss moduli (G″), indicating that the networks are inherently crosslinked (Figure 7A,B). Temperature sweeps reveal the signatures of the DSC-detected phase transitions upon heating from −10 to about 40 °C (Figure 7C,D). The tan δ-peak at around 0 °C also indicates melting of the microphase-separated ordered structure of the vitrimer that was evident from the DSC profiles. We note that due to high moduli, this range of data may be somewhat unreliable. It is followed by an extended rubbery plateau measured up to 180 °C, where tan δ shows a broad and shallow shoulder that may be related to hydrogen-bond rearrangements.

Overall, both vitrimers show rather similar mechanical behavior at elevated temperatures (similar *G*′ of order 0.1 MPa and rather low *G*″), which is remarkable, considering their vastly different microstructures as elucidated by the NMR experiments (Figure 5 and Figure 6). We can conclude that the modulus of Vitrimer **4** is arising from a highly crosslinked single network structure, diluted by about 30% of dangling defects, while Vitrimer **3** with its weaker crosslinked matrix must be significantly reinforced by 30% of the over-cross-linked, more rigid regions, which possibly percolate throughout the sample.

**Figure 8:**
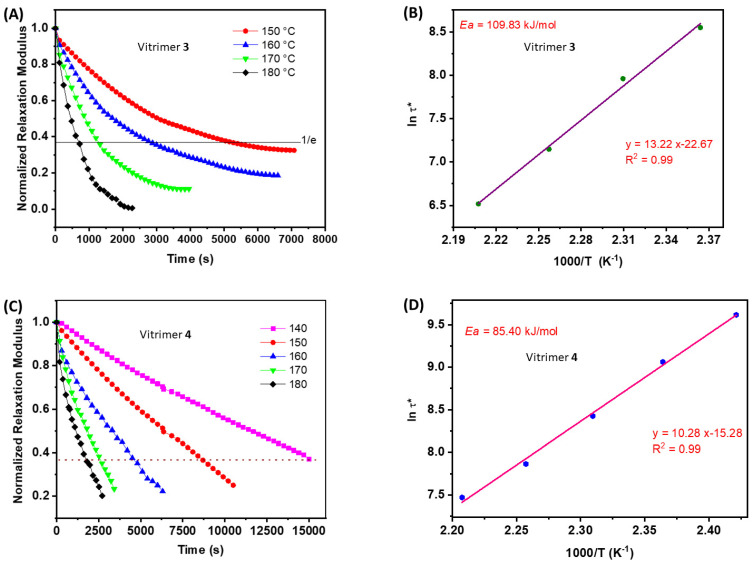
Normalized stress–relaxation curves of vitrimers, **3** (**A**) and **4** (**C**) at different temperature. Arrhenius analysis and the activation energy of vitrimers, **3** (**B**) and **4** (**D**). The dots are data points and lines represent their linear fit.

Stress relaxation measurements of Vitrimers **3** and **4** were also performed in a parallel-plate rheometer after the samples had been heated to the selected measurement temperatures (140–180 °C) for 15 min (Figure 8A,C). The evolution of stress was monitored over time at a given shear strain (1%), which was within the linear viscoelastic range of the samples (Figure S7, Supporting Information). Since the transesterification rate is very slow at a low temperature, the topology of the networks is then practically frozen. At a higher temperature, the vitrimers can rearrange their network topology by transesterification-exchange reactions between the hydroxyl of **2** and the ester bonds of **1**. The curves show significant stress relaxation and the Arrhenius plot was constructed from the time (τ*) corresponding to the G/G_0_ = 0.37 for each temperature scan (Figure 8A,C). *T_v_* is associated with the temperature at which the viscosity reaches 10^12^ Pa s; since *G*′ is about 0.1 MPa for both vitrimers, the corresponding relaxation time would be τ*(*T_v_*) = 10^12^/0.1 × 10^6^ s ≈ 10^7^ s (ln 10^7^ = 16.1 in our activation plots).

It is known that vitrimers with different catalysts have different activation energies (*E_a_*) and different *T_v_* [35]. Since Vitrimer **4** contains more mobile components than Vitrimer **3**, we expect that Vitrimer **4** might have low *E_a_* and *T_v_* values. As expected, Vitrimer **4** displays a lower *E_a_* of 85.40 kJ/mol and a *T_v_* of 29 °C compared with Vitrimer **3** with an *E_a_* of 109.83 kcal/mol and a *T_v_* of 39 °C (Figure 8B,D). These *E_a_* values of our vitrimer were also within the range of known vitrimers [35]. Although we designed vitrimers with lower *T_v_* values by increasing the content of flexible units in the network, microphase separation would reduce the probability of transesterification and thus slow down the relaxation behaviour.

**Figure 9:**
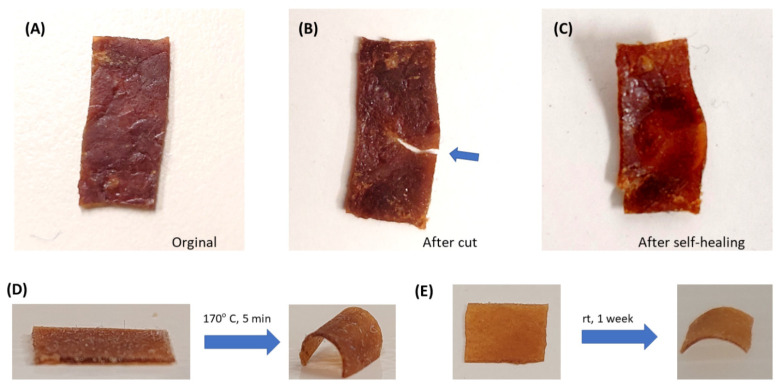
Photographs of Vitrimer **3** before cutting (**A**), after a cut in the middle (**B**) and self-healed after pressed for 5 min under 20 N at 180 °C (**C**). Shape fixing of Vitrimer **3** by applying a pressure at 170 °C (**D**) and room temperature (**E**).

Since macroscopic flow is expected in our vitrimer at elevated temperatures, we also expected self-healing properties of vitrimers. The self-healing ability of Vitrimer **3** was tested by making an incision in the polymer film and then assembling it under a force of 20 N at 180 °C for 5 min (Figure 9A–C). The permanent plastic deformation produced by bond exchange reactions at elevated temperature and by the application of an external force can also be demonstrated by shape change experiments. Vitrimer **3** films were bonded to glass rods with a Teflon tap and then exposed to 170 °C for 5 min or kept at room temperature for one week (Figure 9D,E). After these periods, the films changed to a semi-circular shape that was retained permanently until further heating.

## 4. Conclusions

In summary, two multiphase, low-*T_v_* vitrimers based on flexible polymers such as poly(tri-ethylene glycol sebacate) (**1**) and flexible poly(hydroxyethyl acrylate) (**2**) have been demonstrated for the first time. The copolymeric vitrimer network was synthesized by a facile, solvent-free, thermally induced transesterification in the presence of Zn(OAc)_2_ or Sn(Oct)_2_. The samples exhibit microphase separation at lower temperatures, which can aid in adding shape persistence to the materials, specifically when crystallization is involved. Time-domain-NMR analyses proved that our vitrimers exhibit microphase separation at room temperature and that Vitrimer **4** becomes more homogeneous upon heating compared to Vitrimer **3**. Both, Vitrimer **3** and **4** display a low-*T_v_* close to ambient temperature and Vitrimer **4** displays a lower activation energy and a lower *T_v_* compared to Vitrimer **3** indicative of its higher molecular mobility. Because of the flexibility of the chains and, more importantly, to the abundant hydroxyl groups of Polymer **2** in the network, the materials show typical vitrimer properties such as self-healing and reshaping. Though both vitrimers display low-*T_v_* values, the rate of transesterification was slow at lower temperatures in these vitrimers because of the microphase separation. We therefore we are currently working on vitrimers to reduce issues related to the microphase separation. Since the *T_v_* of these multiphase vitrimers determines the upper service temperature and the lower reprocessing temperature, future work will be directed to achieve a further decrease of *T_v_*, combined with melting transitions at higher temperatures to widen the range of service temperatures. We believe that our results will be of great importance for the development of lower reprocessing temperature vitrimers in the future.

## Data Availability

Data are contained within the article.

## Supplementary Materials

The following supporting information can be downloaded at: https://www.mdpi.com/article/10.3390/polym14122456/s1, Figure S1. GPC analysis of polymer **1** (A) and **2** (B); Figure S2. (A) ^1^H and (B) ^13^C NMRs of polymer **1**; Figure S3. (A) ^1^H and (B) ^13^C NMRs of polymer **2**; Figure S4. ^1^H comparison of Polymer **1** with those of soluble fractions obtained from Vitrimer **3** and **4**. The possible structure of transesterification product obtained from Vitrimer **3** was shown on the top. ^1^H NMR of soluble fraction from Vitrimer **3** with proton integration was shown in the right; Figure S5. ESI-TOF-MS of soluble fraction obtained from Vitrimer **3**. The chemical structure of component identified was shown and it was observed at m/z value of 877.5155. Bottom left figure showed isotopic pattern of peak at 877.5155 (black) which was well matched simulated pattern (red); Figure S6. Free induction decay (FID) and MSE refocused FID analyses of Vitrimer **3** at 100 °C (A) and 180 °C (B); Figure S7. G’ and G’’ plot vs shear strain of Vitrimers **3** (A) and **4** (B) measured at 180 °C. Note that these measurements were done after experiments of Figure 7. The somewhat increased storage modulus may be due to settling of the sample in the rheometer tool and/or aging.
