# Supplementary material for: Design, Synthesis and Characterization of Vitrimers with Low Topology Freezing Transition Temperature
Source: Polymers (Basel). 2022 Jun 16;14(12):2456. doi: 10.3390/polym14122456 (PMC9229622; doi:10.3390/polym14122456)
Supplement: Supplementary file 1 [file polymers-14-02456-s001.zip › polymers-1752236-supplementary.pdf]

polymers

*Supplementary Materials*

# Design, Synthesis and Characterization of Vitrimers with Low Topology Freezing Transition Temperature

**Baiju P. Krishnan [1], Kay Saalwaechter [2], Vico K.B. Adjedje [1] and Wolfgang H. Binder [1,*]**

[1] Macromolecular Chemistry, Division of Technical and Macromolecular Chemistry, Institute of Chemistry, Faculty of Natural Sciences II (Chemistry, Physics and Mathematics), Martin Luther University Halle-Wittenberg, von-Danckelmann-Platz 4, D-06120 Halle, Germany; baijupkrish@gmail.com (B.P.K.); vico.adjedje@chemie.uni-halle.de (V.K.B.A.)

[2] Institut für Physik—NMR, Martin-Luther Universität Halle-Wittenberg, Betty-Heimann-Str. 7, 06120 Halle (Saale), Germany; kay.saalwaechter@physik.uni-halle.de

* Correspondence: wolfgang.binder@chemie.uni-halle.de

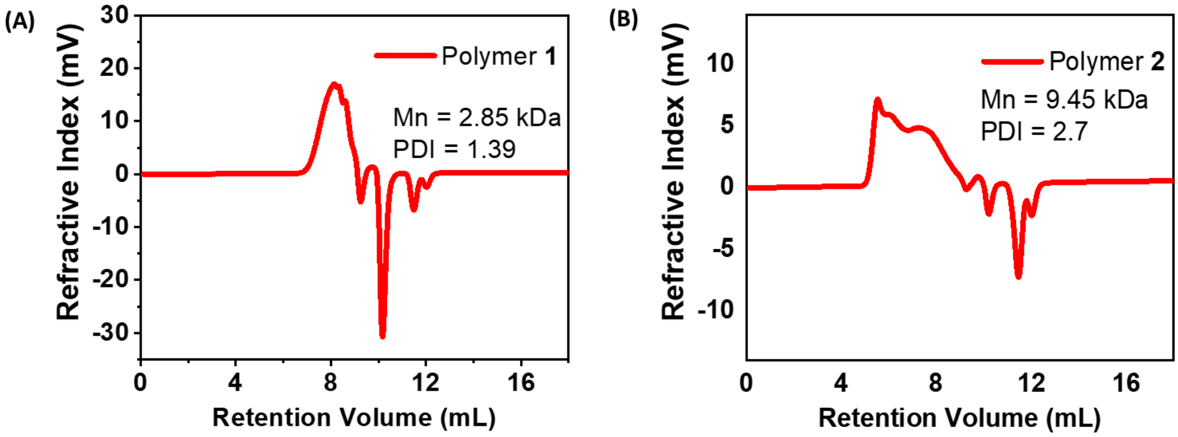


**Figure S1.** GPC analysis of polymer **1** (A) and **2** (B).

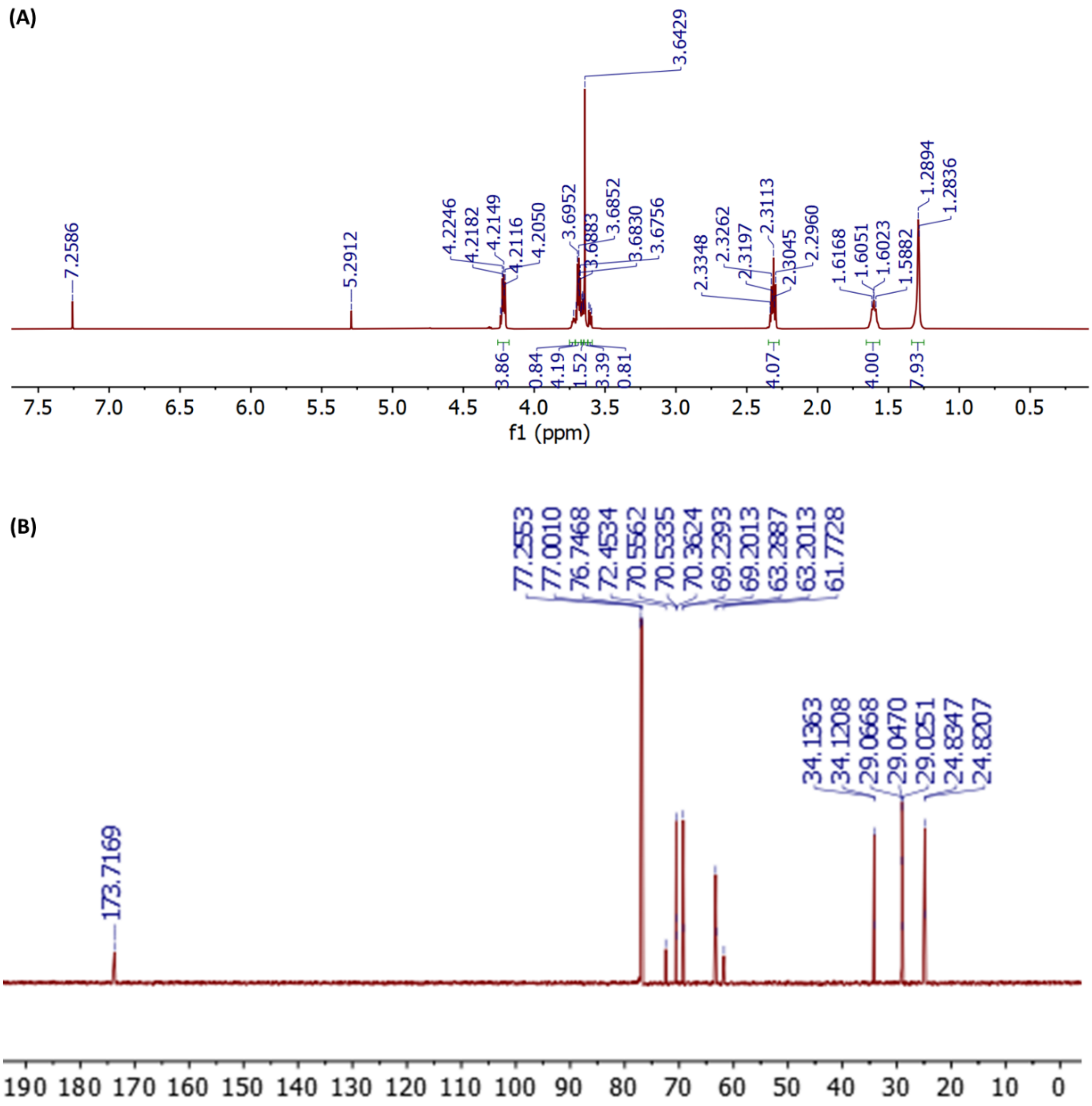


**Figure S2.** (A) $^{1}H$ and (B) $^{13}C$ NMRs of polymer **1**.

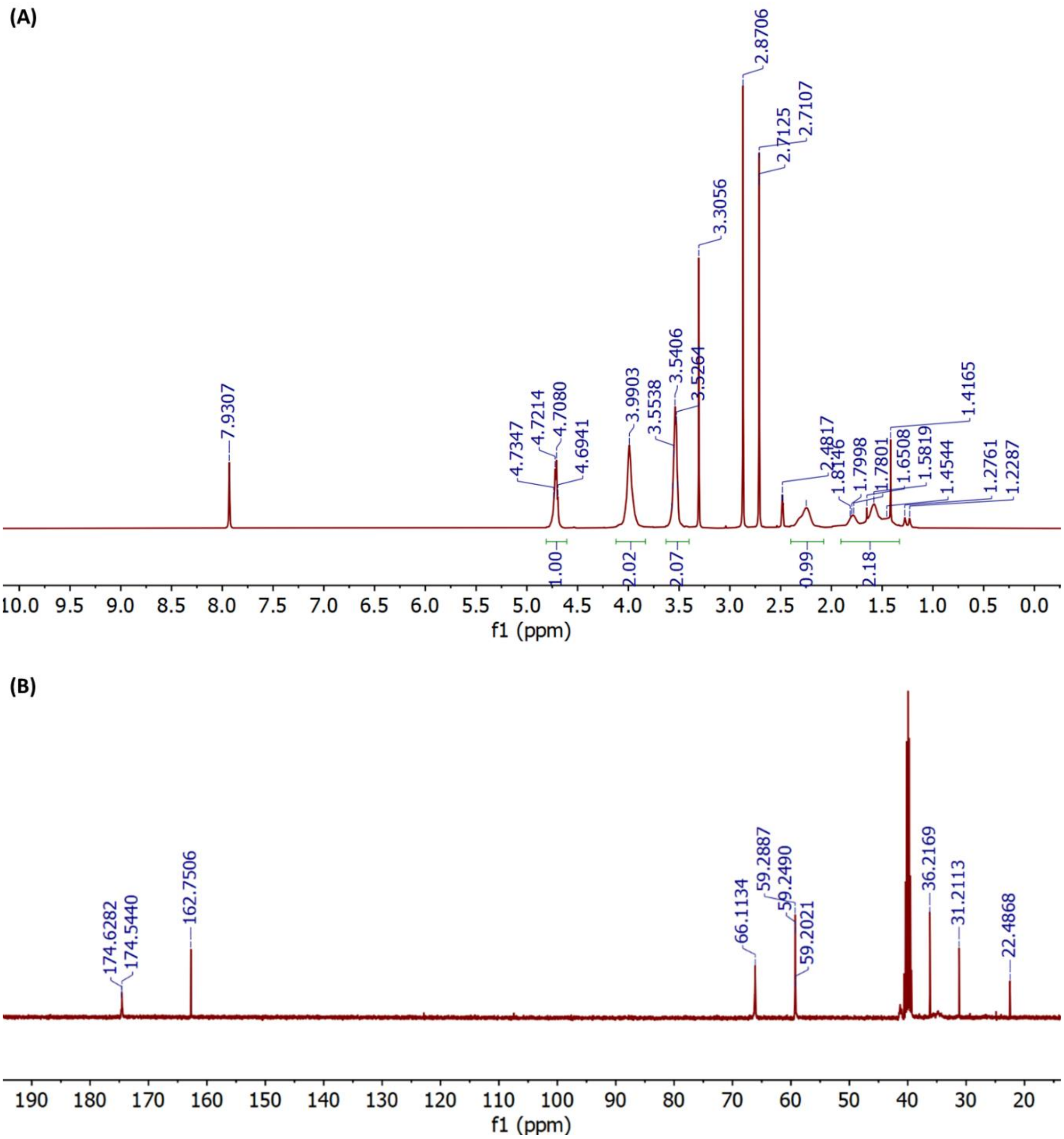


**Figure S3.** (A) $^{1}$H and (B) $^{13}$C NMRs of polymer **2**.

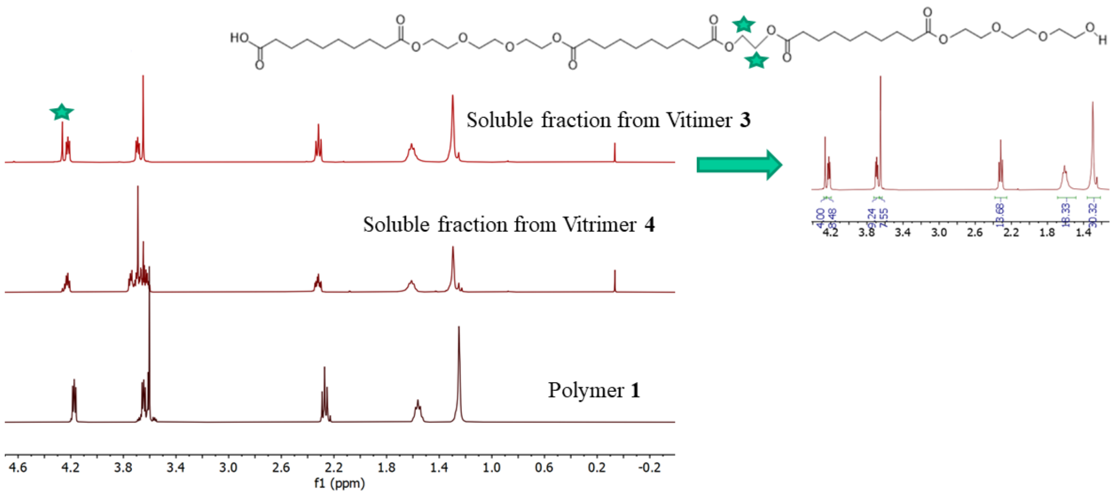

**Figure S4.** ¹H comparison of Polymer **1** with those of soluble fractions obtained from Vitrimer **3** and **4**. The possible structure of transesterification product obtained from Vitrimer **3** was shown on the top. ¹H NMR of soluble fraction from Vitrimer **3** with proton integration was shown in the right.

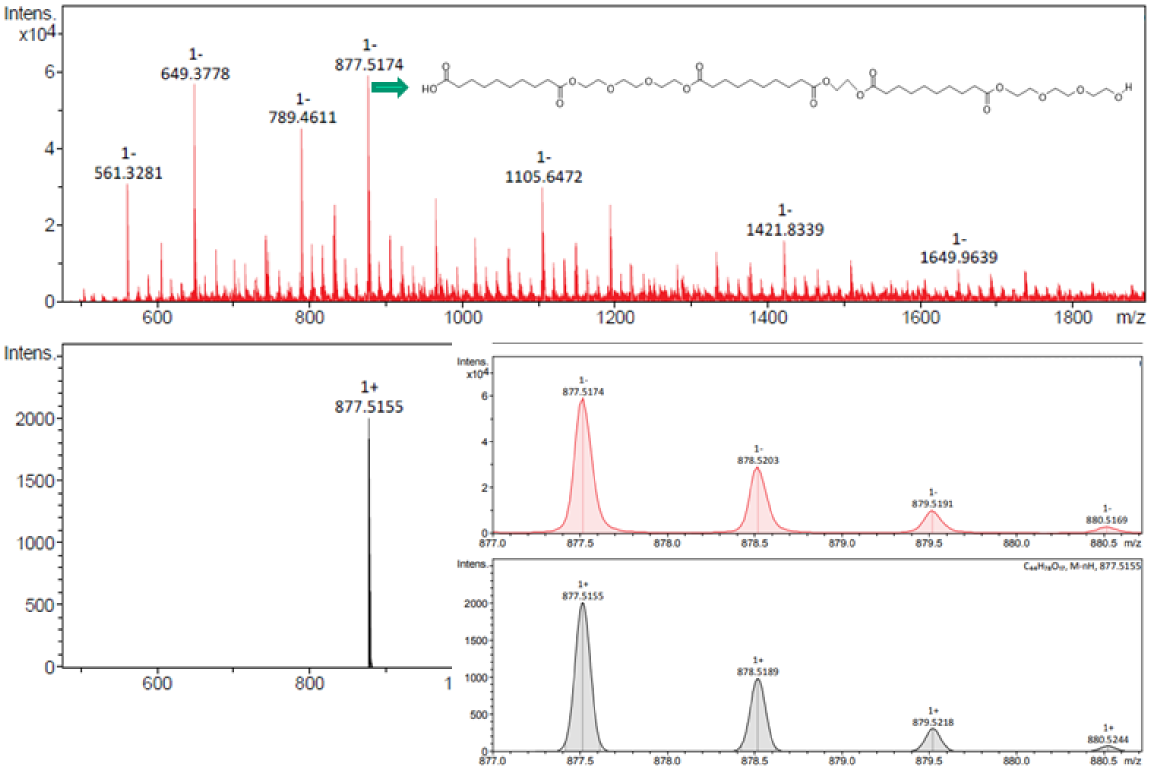


**Figure S5.** ESI-TOF-MS of soluble fraction obtained from Vitrimer **3**. The chemical structure of component identified was shown and it was observed at m/z value of 877.5155. Bottom left figure showed isotopic pattern of peak at 877.5155 (black) which was well matched simulated pattern (red).

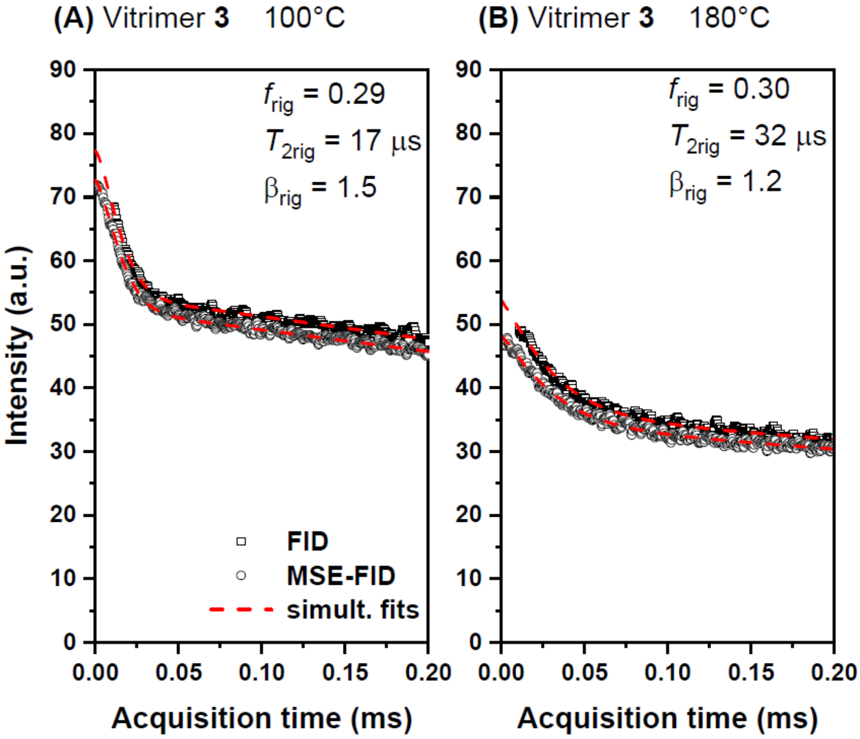


**Figure S6.** Free induction decay (FID) and MSE refocused FID analyses of Vitrimer **3** at 100 °C (A) and 180 °C (B).

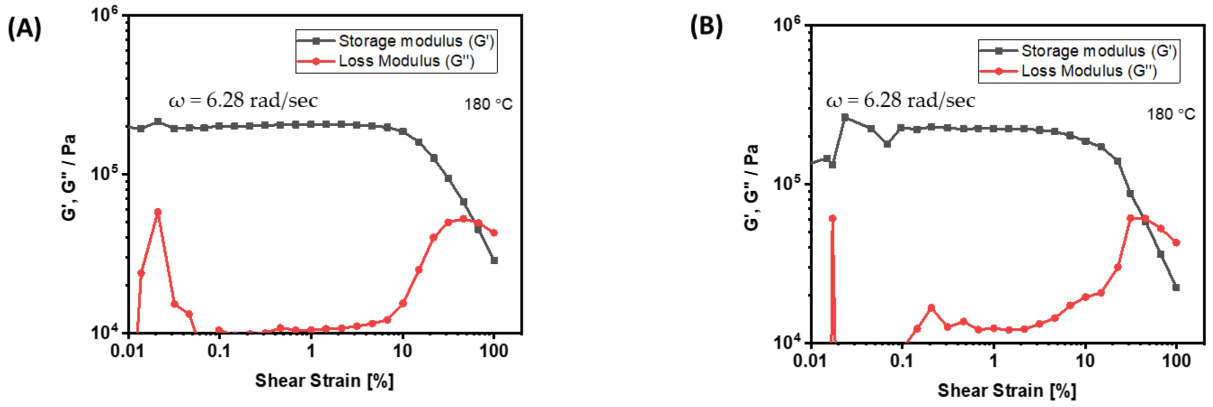


**Figure S7.** G′ and G′′ plot vs shear strain of Vitrimers **3** (A) and **4** (B) measured at 180 °C. Note that these measurements were done after experiments of Figure 7. The somewhat increased storage modulus may be due to settling of the sample in the rheometer tool and/or aging.
